# Association between nineteen dietary fatty acids and hearing thresholds: findings from a nationwide survey

**DOI:** 10.1186/s12944-023-01896-y

**Published:** 2023-08-10

**Authors:** Xiaojin Zhang, Qin Luo, Zhicheng Huang, Xin Xiang

**Affiliations:** 1 Department of Otolaryngology, The Affiliated Nanhua Hospital, Hengyang Medical School, University of South China, Hengyang, China; 2 Department of Otolaryngology, The Second Affiliated Hospital, Hengyang Medical School, University of South China, Hengyang, China

**Keywords:** Fatty acids, Hearing loss, NHANES, Dietary intake, Adults

## Abstract

**Introduction:**

Hearing loss is a prevalent health concern, and dietary factors, such as fatty acid intake, may play a role in its development. The current study aimed to investigate the association between the intake of dietary fatty acids and hearing thresholds among U.S. adults.

**Methods:**

The researchers examined data from the National Health and Nutrition Examination Survey (NHANES), including 7,623 participants with available dietary fatty acid intake and audiometry data. Dietary fatty acid intake was assessed using dietary recalls, and hearing thresholds were measured using pure-tone audiometry. Multivariate linear regression models and smoothing curve fitting were utilized to explore the associations between dietary fatty acid intake and hearing thresholds, adjusting for relevant covariates.

**Results:**

This study reveals a direct association between both low and high frequency pure tone average (PTA) hearing thresholds and the dietary intake of total saturated fatty acids (SFAs) and total polyunsaturated fatty acids (PUFAs). Conversely, the intake of total monounsaturated fatty acids (MUFAs) demonstrates an inverted U-shaped correlation with low-frequency and high-frequency PTA hearing thresholds, having inflection points at 11.91 (energy (%)) and 10.88 (energy (%)), respectively.

**Conclusion:**

Dietary intake of certain fatty acids may influence hearing thresholds in adults.

**Supplementary Information:**

The online version contains supplementary material available at 10.1186/s12944-023-01896-y.

## Introduction

Hearing loss is a widespread public health issue that affects millions of people worldwide [1, 2]. Hearing impairment is a global issue that affects a significant proportion of the population. It has profound effects on communication, social interactions, and overall quality of life [3]. Hearing loss is expected to become more common in the future years as populations age [4], increased exposure to environmental noise [5], and other risk factors [6–8]. Given the substantial societal and economic burden associated with hearing loss, there is a growing interest in identifying modifiable factors, such as diet, that may influence auditory function and potentially mitigate the risk of hearing impairment [9–11].

Fatty acids are one type of dietary component that has received a lot of attention in recent decade because of their numerous implications in human health [12–15]. Fatty acids are integral components of cellular membranes, influencing membrane fluidity, and consequently, the function of membrane-bound proteins, including ion channels and receptors [16, 17]. The mechanotransduction process in hair cells, which are the primary sensory cells that transform sound-induced vibrations into electrical signals within the inner ear, could be influenced by changes in membrane fluidity [18, 19]. Furthermore, fatty acids serve as precursors to bioactive lipid mediators, such as eicosanoids, resolvins, and protectins, which regulate inflammation and oxidative stress – processes known to contribute to the pathogenesis of hearing loss [20, 21]. Research has shown the possible defensive qualities of specific fatty acids, especially omega-3 polyunsaturated fatty acids (PUFAs). These fatty acids could potentially safeguard against hearing loss caused by noise or age due to their anti-inflammatory and antioxidant characteristics [22–24].

While previous investigations have addressed the connection between dietary patterns, nutrient intake, and hearing loss, few studies have specifically focused on the connection between individual fatty acids intake and hearing threshold. Furthermore, existing research largely focuses on specific subtypes of fatty acids, like omega-3 and omega-6 PUFAs, giving less consideration to the wider range of fatty acids that are part of the human diet.

The hypothesis of this study is that dietary intake of certain fatty acids is associated with hearing thresholds in adults. Hence, this study sought to explore the correlation between consumption of various dietary fatty acids and hearing thresholds, utilizing data from the National Health and Nutrition Examination Survey (NHANES). The study considered a total of 19 dietary fatty acids, encompassing saturated, monounsaturated, and polyunsaturated fatty acids.

## Methods

### Study design

**Fig. 1:**
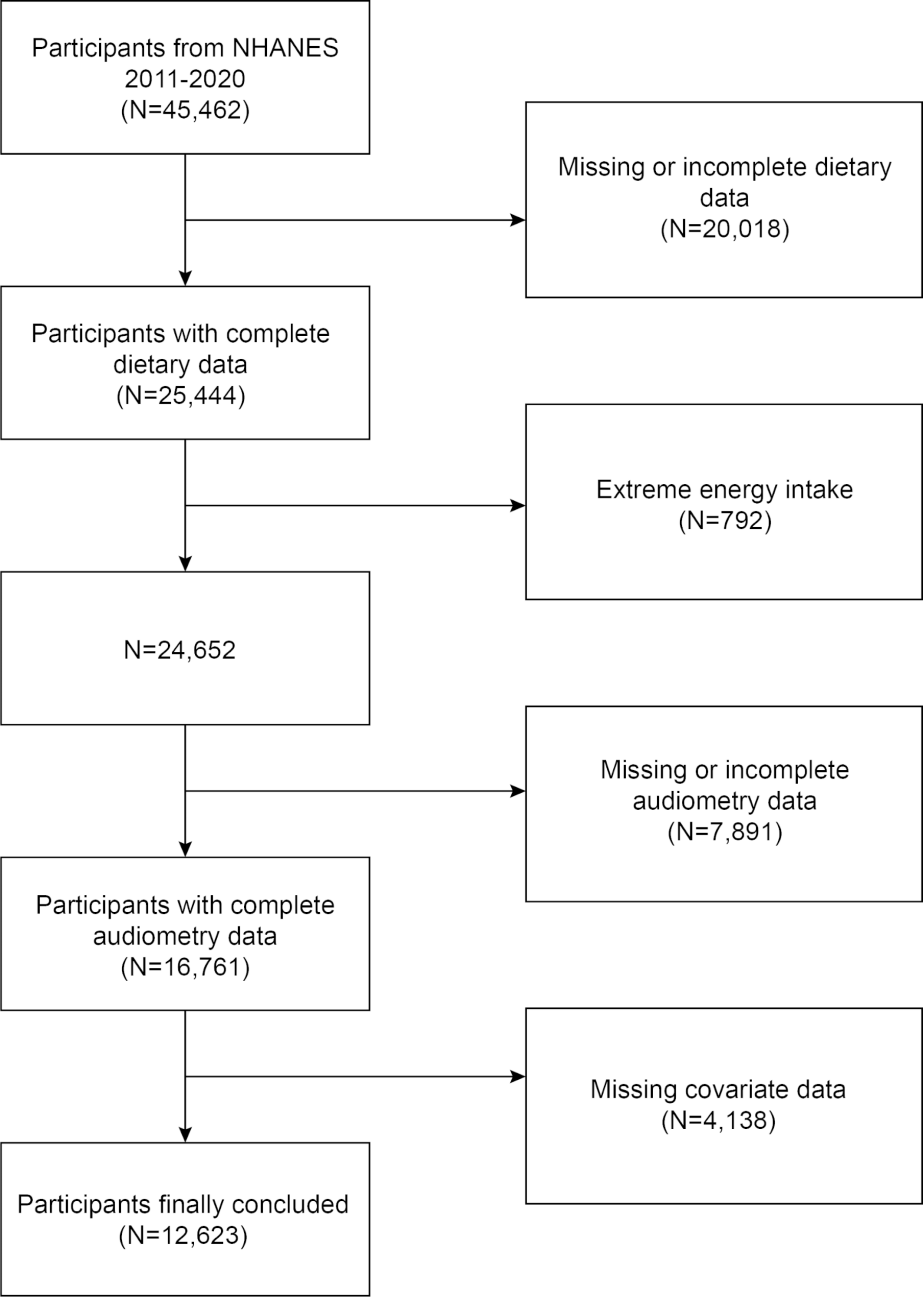
Flow chart of participants selection. Abbreviations: NHANES, National Health and Nutrition Examination Survey

This study employed cross-sectional data from NHANES, a multistage survey that is nationally representative and designed to evaluate the health and nutritional status of adults and children in the United States [25–27]. This analysis was based on data collected between 2011 and 2020, encompassing a total of five NHANES cycles. Excluded 20,018 participants without available dietary fatty acids intake data, 792 participants with extreme total energy intakes, 12,891 participants without complete audiometry data, and 4,138 without covariate data. The research finally included 7,623 adults (Fig. 1).

### Variables

Dietary information were collected using two days dietary recalls, administered in-person and via telephone, respectively [28]. For the purpose of this study, only data from the first 24-hour dietary recall was used to estimate the daily intake of 19 dietary fatty acids. Hearing thresholds were assessed using pure-tone audiometry, audiometric testing was conducted in a soundproof booth by trained examiners, adhering to the American National Standards Institute guidelines. Audiometry, the scientific method of evaluating hearing sensitivity for changes in sound intensity and pitch, primarily employs hearing thresholds as a key metric. The hearing threshold represents the softest sound an individual can detect at least half of the time. It’s crucial to understand that a heightened hearing threshold signifies decreased hearing ability, while a reduced threshold indicates improved hearing acuity. This principle arises from the measurement of hearing thresholds in decibels, a unit of sound intensity, where a higher dB level corresponds to the ability to perceive only louder sounds [29]. Audiology uses the pure-tone average (PTA), a calculated figure, to gauge the extent of hearing loss. In this research, the low-frequency PTA was computed as the mean of the hearing thresholds at 500, 1000, and 2000 Hz, while the high-frequency PTA was the average of the hearing thresholds at 4000, 6000, and 8000 Hz. These frequency ranges were selected as they best represent the frequency range of human speech and are most pertinent to speech comprehension. It’s noteworthy that increased PTA values equate to poorer hearing ability [30].

### Statistical Analysis

All statistical procedures were conducted using R software and Empowerstats. The demographic traits of the study participants were assessed via chi-square tests for categorical variables and t-tests for continuous variables, differentiated by gender. A univariate logistic regression was leveraged to investigate the relationship between covariates and hearing thresholds. In order to probe the linear association between dietary fatty acids and hearing thresholds, multivariate linear and logistic regression models were put to use. For curve smoothing, the penalized spline method was adopted, and a two-segment linear regression model was implemented to study the possible nonlinear correlation between fatty acid intake and hearing thresholds [31–33]. The models took into account relevant covariates such as age, sex, race/ethnicity, education and diabetes. Results were presented as beta coefficients accompanied by 95% confidence intervals (CIs) for linear regression models. A two-sided P-value less than 0.05 was considered to indicate statistical significance.

## Results

### Baseline characteristics

The research included 7,623 participants, with a mean age (SD) of 44.15 ± 12.98 years, composed of 3,652 (47.91%) males and 3,971 (52.09%) females. The low and high-frequency PTA hearing thresholds were recorded at 7.51 ± 6.75 dB and 21.56 ± 17.43 dB, respectively. The average proportions of energy derived from saturated fatty acids (SFAs), monounsaturated fatty acids (MUFAs), and PUFAs were 11.55%, 12.60%, and 9.25%. Male participants were observed to have a higher MUFA intake and a higher high-frequency PTA in comparison to female participants (Table 1).

**Table 1:** Basic characteristics of participants by gender among U.S. adults

Characteristics	Male (3,652)	Female (3,971)	*P*-value
Age (years)	44.51 ± 12.92	43.84 ± 13.10	0.511
Race/ethnicity, (%)			0.203
Non-Hispanic White	65.51	65.34	
Non-Hispanic Black	14.59	14.75	
Mexican American	6.98	9.45	
Other race/multiracial	16.92	11.46	
Education level, (%)			0.013
Less than high school	17.92	18.82	
High school	24.85	16.31	
More than high school	57.23	64.87	
Diabetes, (%)			0.091
Yes	10.51	11.22	
No	89.49	88.78	
Low-frequency PTA (dB)	7.31 ± 6.80	7.95 ± 7.21	0.088
High-frequency PTA (dB)	22.31 ± 18.80	18.99 ± 15.27	0.001
Dietary intake			
Energy (kcal/day)	2267.01 ± 919.59	2050.58 ± 1009.15	< 0.001
Total SFA(energy (%))	11.83 ± 3.34	11.42 ± 3.71	0.218
SFA 4:0 (Butanoic)	0.21 ± 0.16	0.20 ± 0.15	0.834
SFA 6:0 (Hexanoic)	0.14 ± 0.11	0.13 ± 0.10	0.907
SFA 8:0 (Octanoic)	0.13 ± 0.10	0.12 ± 0.10	0.175
SFA 10:0 (Decanoic)	0.22 ± 0.16	0.22 ± 0.16	0.985
SFA 12:0 (Dodecanoic)	0.41 ± 0.62	0.39 ± 0.66	0.117
SFA 14:0 (Tetradecanoic)	0.96 ± 0.57	0.96 ± 0.56	0.871
SFA 16:0 (Hexadecanoic)	6.43 ± 1.76	6.41 ± 1.85	0.140
SFA 18:0 (Octadecanoic)	2.57 ± 0.93	2.99 ± 0.93	0.092
Total MUFA (energy (%))	12.79 ± 3.51	12.53 ± 3.30	0.049
MUFA 18:0 (Hexadecenoic)	0.58 ± 0.26	0.55 ± 0.28	0.011
MUFA 18:1 (Octadecenoic)	11.99 ± 3.30	11.77 ± 3.43	0.057
MUFA 20:1 (Eicosenoic)	0.18 ± 0.10	0.19 ± 0.11	0.411
MUFA 22:1 (Docosenoic)	0.04 ± 0.05	0.04 ± 0.06	0.286
Total PUFA(energy (%))	9.25 ± 3.69	8.82 ± 3.45	0.088
PUFA 18:2 (Octadecadienoic)	7.99 ± 3.80	7.88 ± 3.71	0.191
PUFA 18:3 (Octadecatrienoic)	0.89 ± 0.50	0.86 ± 0.43	0.056
PUFA 18:4 (Octadecatetraenoic)	0.02 ± 0.02	0.02 ± 0.02	0.513
PUFA 20:4 (Eicosatetraenoic)	0.09 ± 0.06	0.09 ± 0.06	0.111
PUFA 20:5 (Eicosapentaenoic)	0.04 ± 0.07	0.04 ± 0.08	0.532
PUFA 22:5 (Docosapentaenoic)	0.03 ± 0.02	0.03 ± 0.03	0.168
PUFA 22:6 (Docosahexaenoic)	0.07 ± 0.12	0.06 ± 0.12	0.535

Mean ± SD for continuous variables: the P value was calculated by the weighted linear regression model. (%) for categorical variables: the P value was calculated by the weighted chi-square test

PTA, Pure-tone average; SFA, saturated fatty acid; MUFA, monounsaturated fatty acid; PUFA, polyunsaturated fatty acid

Table 2 shows the relationship between all covariates and hearing thresholds. Females had lower High-frequency PTA compared to males; other races tended to have higher hearing thresholds compared to non-Hispanic whites, although these numbers were not statistically significant; participants 60 years and older had higher hearing thresholds than those under 60 years of age; participants with higher education levels tended to have lower hearing thresholds; and, participants with diabetes participants tended to have higher hearing thresholds than other participants.

**Table 2:** Weighted univariate logistic regression analysis of factors associated with hearing thresholds

Exposure	Low-frequency PTA β (95%CI)	High-frequency PTA β (95%CI)
Sex		
Male	0 (ref)	0 (ref)
Female	0.61 (-2.38, 4.43)	-4.16 (-10.55, -2.01)*
Race/ethnicity		
Non-Hispanic White	0 (ref)	0 (ref)
Non-Hispanic Black	0.25 (-0.32, 0.86)	1.76 (-2.33, 5.92)
Mexican American	0.11 (-0.31, 0.52)	1.00 (-1.38, 3.99)
Other race/multiracial	0.35 (-0.26, 0.90)	2.52 (-3.15, 7.01)
Education		
Less than high school	0 (ref)	0 (ref)
High school	-0.35 (-0.51, -0.20) *	-1.56 (-3.02, -0.08) *
More than high school	-1.21 (-1.87, -0.60) *	-3.30 (-5.81, -0.68) *
Diabetes		
Yes	0 (ref)	0 (ref)
No	-1.41 (-2.53, -0.30)*	-5.08 (-9.33, -0.88)*
Age, years < 60 ≥ 60	0 (ref) 2.21 (0.16, 4.58)*	0 (ref) 5.71 (1.09, 10.25)*
Energy (kcal/day)	0.00 (0.00, 0.01)*	0.00 (0.00, 0.01)*
Total SFA (energy (%))	0.05 (0.01, 0.09)*	0.13 (-0.02, 0.29)
Total MUFA (energy (%))	0.03 (-0.03, 0.06)	0.15 (-0.30, 0.61)
Total PUFA (energy (%))	0.07 (-0.10, 0.24)	0.09 (-0.15, 0.33)

Abbreviations: 95% CI, 95% confidence interval; PTA, Pure-tone average; Hs-CRP, High-Sensitivity C-Reactive Protein; SFA, saturated fatty acid; MUFA, monounsaturated fatty acid; PUFA, polyunsaturated fatty acid. * indicates p < 0.05, indicating statistical significance

### Association between Fatty Acids and Hearing Thresholds

A comparable link was found between high-frequency PTA and total SFA and total PUFA, while no statistically significant association was observed with total MUFA (Tables 3 and 4). Examination of the relationship between high-frequency PTA and fatty acid subclasses showed that the positive correlation between high-frequency PTA and SFAs was primarily due to SFA 4:0, SFA 6:0, SFA 8:0, and SFA 10:0. Among these, SFA 4:0 had the most significant effect, resulting in a significant increase of 1.71 dB [1.71 (0.09, 3.43)] in high-frequency PTA for each 1% increase in the proportion of total energy intake. The positive correlation between high-frequency PTA and PUFA was mainly derived from PUFA 20:4, PUFA 20:5, PUFA 22:5, and PUFA 22:6. Of these, PUFA 22:6 had the most substantial effect, with a significant rise in high-frequency PTA of 6.40 dB [6.40 (1.51, 10.87)] for each 1% increase in total energy intake.

**Table 3:** Multivariate linear regression analysis of the association between dietary fatty acids and Low-frequency PTA.

Fatty acids	Continuous	Q1	Q2	Q3	Q4	*P* for trend
(energy (%))	β (95% CI)		β (95% CI)	β (95% CI)	β (95% CI)	
Total SFAs	0.04 (0.01, 0.08)*	0 (ref)	0.26 (-0.34, 0.51)	0.45 (-0.15, 1.07)	0.48 (0.14, 0.99)*	0.035
SFA 4:0	0.52 (0.02, 1.04)*	0 (ref)	0.52 (0.14, 1.16)*	0.34 (-0.02, 0.86)	0.47 (-1.06, 2.22)	0.031
SFA 6:0	0.56 (0.08, 1.61)*	0 (ref)	0.12 (-0.03, 0.35)	0.29 (-0.36, 1.23)	0.32 (-0.22, 0.89)	0.012
SFA 8:0	0.19 (-0.08, 0.45)	0 (ref)	-0.01 (-0.72, 0.58)	0.42 (0.04, 0.93)*	0.54 (-0.08, 1.10)	0.065
SFA 10:0	-0.01 (-2.00, 0.78)	0 (ref)	0.28 (-0.37, 0.93)	-0.09 (-0.74, 0.55)	-0.11 (-0.75, 0.53)	0.408
SFA 12:0	-0.01 (-0.57, 0.34)	0 (ref)	-0.03 (-0.68, 0.62)	-0.63 (-1.28, 0.01)	-0.46 (-1.10, 0.17)	0.253
SFA 14:0	-0.09 (-0.46, 0.30)	0 (ref)	0.34 (-0.31, 0.99)	-0.19 (-0.83, 0.45)	-0.13 (-0.61, 0.68)	0.398
SFA 16:0	-0.13 (0.02, 0.25)	0 (ref)	-0.23 (-0.90, 0.43)	0.26 (-0.39, 0.90)	0.43 (-0.20, 1.06)	0.078
SFA 18:0	-0.32 (-1.07, 0.55)	0 (ref)	-0.43 (-1.21, 0.07)	0.70 (0.06, 1.34)*	0.79 (0.16, 1.42)*	0.213
Total MUFAs	0.04 (-0.02, 0.10)	0 (ref)	-0.48 (-1.12, 0.17)	-0.15 (-0.79, 0.48)	0.25 (-0.38, 0.87)	0.107
MUFA 18:0	0.52 (-0.24, 1.26)	0 (ref)	0.13 (-0.52, 0.78)	0.37 (-0.27, 1.01)	0.23 (-0.40, 0.85)	0.407
MUFA 18:1	0.05 (-0.04, 0.09)	0 (ref)	-0.54 (-1.19, 0.12)	-0.17 (-0.81, 0.46)	0.28 (-0.35, 0.91)	0.188
MUFA 20:1	-1.01 (-3.10, 1.01)	0 (ref)	0.05 (-0.60, 0.68)	-0.07 (-0.70, 0.56)	0.17 (-0.46, 0.80)	0.599
MUFA 22:1	-0.57 (-4.71, 3.61)	0 (ref)	0.18 (-0.46, 0.81)	-0.12 (-0.74, 0.53)	0.26 (-0.38, 0.89)	0.469
Total PUFAs	0.06 (0.03, 0.09)*	0 (ref)	-0.38 (-1.02, 1.21)	-0.01 (-0.95, 2.31)	0.41 (-0.61, 0.65)	0.143
PUFA 18:2	0.03 (-0.05, 0.09)	0 (ref)	-0.39 (-1.03, 0.25)	-0.26 (-0.89, 0.37)	0.02 (-0.60, 0.64)	0.713
PUFA 18:3	-0.07 (-0.58, 0.41)	0 (ref)	0.03 (-0.60, 0.67)	0.03 (-0.60, 0.66)	-0.01 (-0.63, 0.62)	0.621
PUFA 18:4	-4.64 (-12.05, 5.95)	0 (ref)	0.63 (0.02, 1.25)*	0.52 (-0.10, 1.14)	0.15 (-0.49, 0.78)	0.420
PUFA 20:4	2.24 (-0.66, 4.09)	0 (ref)	-0.24 (-0.86, 0.38)	0.16 (-0.46, 0.79)	0.91 (-0.75, 3.54)	0.996
PUFA 20:5	1.12 (-0.89, 3.69)	0 (ref)	-0.22 (-0.84, 0.40)	0.10 (-0.53, 0.73)	0.80 (-1.43, 4.16)	0.050
PUFA 22:5	1.65 (0.36, 3.56)*	0 (ref)	0.17 (-0.46, 0.80)	0.07 (-0.56, 0.70)	0.67 (0.11, 1.32)*	0.023
PUFA 22:6	1.33 (0.25, 3.09)*	0 (ref)	0.06 (-0.53, 0.68)	0.21 (-0.45, 0.81)	0.94 (0.33, 2.05)*	0.015

Models were adjusted for sex, age, race, education, diabetes, and energy. Continuous, Ln-transformed concentration of fatty acids; Q, quartile; 95% CI, 95% confidence interval; PTA, Pure-tone average; SFA, saturated fatty acid; MUFA, monounsaturated fatty acid; PUFA, polyunsaturated fatty acid. * indicates p < 0.05, indicating statistical significance.

**Table 4:** Multivariate linear regression analysis of the association between dietary fatty acids and High-frequency PTA.

Fatty acids	Continuous	Q1	Q2	Q3	Q4	*P* for trend
(energy (%))	β (95% CI)		β (95% CI)	β (95% CI)	β (95% CI)	
Total SFAs	0.63 (0.06, 1.20)*	0 (ref)	0.56 (0.34, 1.02)*	0.92 (0.15, 1.67)*	1.45 (0.11, 3.09)*	< 0.001
SFA 4:0	1.71 (0.09, 3.43)*	0 (ref)	0.52 (0.14, 1.16)*	1.15 (-0.02, 3.86)	3.49 (1.06, 5.41)*	< 0.001
SFA 6:0	1.06 (-0.28, 3.24)	0 (ref)	-0.01 (-0.33, 0.80)	0.26 (-0.03, 0.73)	1.32 (-0.21, 1.63)	0.016
SFA 8:0	0.17 (-0.08, 0.45)	0 (ref)	-0.01 (-0.72, 0.58)	0.42 (0.04, 0.93)	0.54 (-0.08, 1.10)	0.066
SFA 10:0	0.01 (-1.06, 0.84)	0 (ref)	-0.28 (-0.37, 0.60)	-0.25 (-2.55, 2.02)	0.41 (-3.44, 4.59)	0.103
SFA 12:0	-0.61 (-1.35, 0.38)	0 (ref)	-0.01 (-1.58, 3.21)	-0.24 (-1.28, 1.05)	-0.58 (-4.10, 5.11)	0.661
SFA 14:0	1.01 (-4.11, 5.36)	0 (ref)	0.16 (-1.31, 3.01)	0.11 (-1.47, 2.48)	2.01 (-5.61,9.23)	0.276
SFA 16:0	-0.81 (-2.88, 4.10)	0 (ref)	-0.17 (-0.90, 0.72)	-1.74 (-2.15, 0.81)	-1.36 (-2.22, 0.81)	0.470
SFA 18:0	-0.30 (-0.07, 1.00)	0 (ref)	-0.01 (-1.15, 1.06)	-0.21 (-0.76, 0.30)	-0.16 (-2.10, 1.48)	0.605
Total MUFAs	-0.03 (-0.71, 0.63)	0 (ref)	-0.10 (-2.17, 3.13)	-0.15 (-2.69, 2.00)	-0.31 (-3.18, 3.89)	0.093
MUFA 18:0	-1.11 (-4.61, 7.17)	0 (ref)	0.15 (-0.12, 0.48)	0.30 (-0.71, 1.92)	-1.03 (-4.12, 2.22)	0.676
MUFA 18:1	0.10 (-2.23, 3.01)*	0 (ref)	-0.50 (-4.15, 3.57)	1.31 (-0.21, 1.89)	0.41 (-1.55, 1.84)	0.558
MUFA 20:1	-3.31 (-7.92, 1.06)	0 (ref)	-1.01 (-4.14, 0.88)	-2.55 (-6.72, 1.40)	-3.11 (-9.21, 4.87)	0.035
MUFA 22:1	-1.15 (-5.91, 4.81)	0 (ref)	0.21 (-2.41, 2.88)	-1.11 (-3.04, 1.06)	-1.16 (-5.30, 2.77)	0.138
Total PUFAs	1.32 (0.27, 2.73)*	0 (ref)	0.16 (0.02, 0.36)*	0.49 (0.16, 1.15)*	2.11 (0.15, 4.21)*	< 0.001
PUFA 18:2	1.02 (0.04, 2.06)*	0 (ref)	1.42 (0.08,2.34)*	1.26 (-0.19, 3.02)	4.62 (-1.21, 9.33)	0.055
PUFA 18:3	2.37 (-1.28, 4.48)	0 (ref)	2.03 (-1.35, 4.59)	1.90 (-1.23, 4.31)	1.05 (-0.66, 2.21)	0.417
PUFA 18:4	0.53 (-1.07, 3.32)	0 (ref)	-0.08 (-1.54, 1.89)	1.50 (-3.17, 7.01)	1.41 (-2.15, 5.50)	0.522
PUFA 20:4	3.05 (0.89, 6.68)*	0 (ref)	1.45 (0.11, 3.41)*	3.51 (-0.40, 8.12)	5.56 (1.35, 12.08)*	0.027
PUFA 20:5	3.69 (1.31, 5.85)*	0 (ref)	1.22 (0.01, 2.28)*	4.11 (1.20, 7.31)*	8.10 (2.02, 15.86)*	< 0.001
PUFA 22:5	5.15 (1.16, 11.33)*	0 (ref)	3.51 (1.06, 7.25)*	6.00 (1.41, 13.20)*	11.21 (3.36, 20.98)*	0.002
PUFA 22:6	6.40 (1.51, 10.87)*	0 (ref)	4.02 (0.50, 8.15)*	4.80 (1.12, 9.57)*	12.11 (1.97, 22.62)*	< 0.001

Models were adjusted for sex, age, race, education, diabetes, and energy. Continuous, Ln-transformed concentration of fatty acids; Q, quartile; 95% CI, 95% confidence interval; PTA, Pure-tone average; SFA, saturated fatty acid; MUFA, monounsaturated fatty acid; PUFA, polyunsaturated fatty acid. * indicates p < 0.05, indicating statistical significance.

**Table 5:** Threshold effect analysis of total MUFA on hearing thresholds by two-piecewise linear regression model

Total MUFA (energy (%))	Fitting by the standard linear model	Fitting by the two-piecewise linear model			
		Inflection point (K)	< K-segment effect	> K-segment effect	Log likelihood ratio
Low-frequency PTA	0.04 (-0.02, 0.10)	11.91	0.21 (0.03, 0.40)	-0.07 (-0.25, 0.11)	< 0.001
High-frequency PTA	-0.03 (-0.71, 0.63)	10.88	0.12 (0.02, 0.23)	-0.13 (-0.26, 0.00)	< 0.001

Models were adjusted for sex, age, race, education, diabetes and energy. Ln-transformed concentration of fatty acids; Q, quartile; 95% CI, 95% confidence interval; PTA, Pure-tone average; SFA, saturated fatty acid; MUFA, monounsaturated fatty acid; PUFA, polyunsaturated fatty acid

**Fig. 2:**
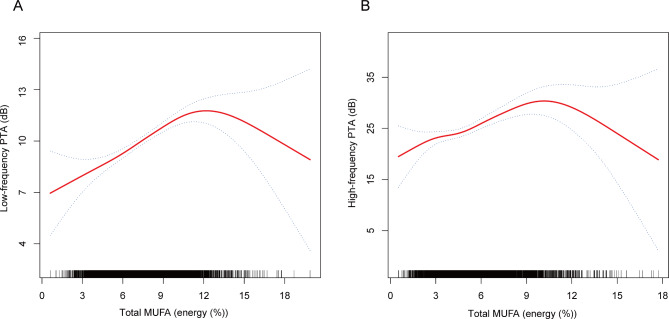
The nonlinear associations between MUFAs and hearing thresholds. The solid red line represents the smooth curve fit between variables. Blue bands represent the 95% of confidence interval from the fit. **(A)** Total MUFA and low-frequency PTA; **(B)** Total MUFA and high-frequency PTA. Abbreviations: MUFA, monounsaturated fatty acid; Pure-tone average (PTA)

Given the absence of a significant linear association between total MUFA intake and both low-frequency PTA and high-frequency PTA, a further exploration of the non-linear relationship between total MUFA intake and hearing thresholds was undertaken (Table 5). Figure 2 visually depicts the nonlinear correlation between fatty acid intake and hearing thresholds, as determined using the penalized spline method. The findings demonstrated an inverted U-shaped correlation between total MUFA and both low-frequency PTA and high-frequency PTA, with inflection points at 11.91 (energy (%)) and 10.88 (energy (%)), respectively (log-likelihood ratio < 0.01).

## Discussion

This research provide evidence of a positive relationship between both low and high-frequency PTA and the intake of total SFA and PUFA. It is important to clarify that a positive association in this context indicates that higher intake of these fatty acids is associated with higher hearing thresholds, which signifies poorer hearing ability. In light of theses findings, higher intake of SFA and PUFA are associated with higher hearing thresholds, suggesting a potential detrimental effect. While these are preliminary findings lacking causality, they suggest that adjusting the intake of these specific fatty acids could influence hearing thresholds. However, it is important to stress that these findings should not be directly used to make clinical recommendations without further investigation. Furthermore, a non-linear, inverted U-shaped association between low and high-frequency PTA and MUFA consumption were observed. The curves suggest that moderate intake of MUFAs may be associated with poorer hearing ability, while both low and high intakes may be beneficial. Specifically, the bottom end of the curve, representing low and high intake levels, was associated with better hearing thresholds, indicating a potential beneficial effect. The top of the U-curve represents moderate intake levels, which are associated with the highest hearing thresholds, indicating potential adverse effects on hearing. However, these are preliminary findings and should be interpreted with caution.

This study builds on previous population-based studies that have examined the relationship between the intake of different types of dietary fatty acids and hearing loss [34–39]. Gopinath et al. explored the connection among omega-3 fatty acids and the incidence of deafness in a cohort of over 3,000 participants aged above 50, demonstrating that higher intake of long-chain n-3 PUFAs might reduce hearing loss [34]. A prospective study based on Dutch elderly individuals revealed a negative association between plasma n-3 PUFAs and hearing loss [36]. Two other population-based studies from the United States also support a negative association between PUFAs and lower hearing loss [35, 37]. Echoing previous studies, these findings suggest that specific dietary PUFAs could have a dual effect, being both beneficial and harmful to human auditory health. As an example, Dullemeijer et al. noted a negative correlation between plasma n-3 PUFAs and low-frequency hearing levels [38], while another longitudinal observational cohort study discovered no apparent connection between PUFAs and auditory function [39]. This research expands on these prior studies by examining a broader range of dietary fatty acids, providing a more comprehensive understanding of their potential impact on hearing.

Few research have looked at the relationship between dietary SFAs and MUFAs and hearing [40]. A recent UK biobank-based cohort study investigated the association between total SFAs, MUFAs and PUFA intake and disabling hearing impairment in UK adults [38]. Their results suggest that the intake of polyunsaturated fatty acids is associated with a lower incidence of hearing loss, that replacing 5% of total SFA energy intake with an equivalent amount of dietary polyunsaturated fatty acids may help delay hearing loss, and that the association between SFA and MUFA with hearing remains uncertain. Further investigated these issues in US adults, and these findings indicate that the association between dietary fatty acid subgroups and hearing varies and that the differing effects of various types of fatty acid intake on hearing may account for the discrepancies in results across studies. Additionally, the results of the smoothed curve fit suggest that the association between total MUFA intake and hearing is nonlinear and that inflection points exist. This implies that controlling total MUFA intake within a specific range may be beneficial for hearing, while exceeding the inflection point may yield the opposite effect.

The current study’s outcomes are in agreement with prior research highlighting the advantageous impacts of certain fatty acids on hearing levels [41–43]. It’s been theorized that n-3 PUFAs could enhance hearing by sustaining adequate cochlear blood supply through various mechanisms, such as reducing triglycerides, chronic inflammation, and possessing anti-inflammatory and anti-atherothrombotic properties [44–46]. Likewise, dietary n-6 PUFA has been found to improve endothelial function and chronic inflammation [43]. Moreover, this research expands on these insights by revealing that SFAs have a positive correlation with hearing thresholds. This finding implies that the association between SFAs and hearing levels may be more intricate than previously perceived and might vary due to SFA subclass intake. As suggested by prior studies, the potential beneficial impacts of SFA, particularly short-chain fatty acid intake, on hearing might arise from their role in modulating inflammation, the immune system, and associated G protein-coupled receptors [47, 48].

### Study Strengths and Limitations

The current investigation possesses several strengths, encompassing the extensive, nationally representative sample and the thorough evaluation of a broad array of dietary fatty acids [49, 50]. Nevertheless, some limitations warrant consideration. First and foremost, the study’s cross-sectional design prevents the drawing of conclusions about causality. Secondly, dietary data were procured through self-report, which may be subject to recall bias and measurement inaccuracies. Third, the significant amount of missing data in the larger NHANES dataset. Many participants were removed from the analysis due to missing dietary information, hearing loss, and/or covariates. It remains unclear whether there was any systematic or non-random reason why certain NHANES participants were missing certain data. In particular, for the self-reported dietary data, there could be non-random reasons why some participants did not complete this task, such as time constraints, lack of interest, or misunderstanding of the questions. The missing data might have introduced some degree of bias into the results, and this should be taken into account when interpreting these findings. Finally, residual confounding due to unmeasured or inadequately assessed covariates cannot be discounted.

## Conclusion

The findings from the present study suggest that dietary intake of certain fatty acids may influence hearing thresholds in adults. If confirmed by future studies, these findings could lead to new dietary recommendations for the prevention or management of hearing loss.

### Electronic supplementary material

Below is the link to the electronic supplementary material.

Supplementary Material 1

Supplementary Material 2

## Data Availability

The survey data are publicly available on the internet for data users and researchers throughout the world ( www.cdc.gov/nchs/nhanes/ ).

## List of abbreviations

PTA: Pure-tone average
NHANES: National Health and Nutrition Examination Survey
CIs: Confidence intervals
SFAs: Saturated fatty acids
MUFAs: Monounsaturated fatty acids
PUFAs: Polyunsaturated fatty acids
SCFAs: Short-chain fatty acids
Butanoic: SFA 4:0
Hexanoic: SFA 6:0
Octanoic: SFA 8:0
Decanoic: SFA 10:0
Dodecanoic: SFA 12:0
Tetradecanoic: SFA 14:0
Hexadecanoic: SFA 16:0
Octadecanoic: SFA 18:0
Hexadecenoic: MUFA 18:0
Octadecenoic: MUFA 18:1
Eicosenoic: MUFA 20:1
Docosenoic: MUFA 22:1
Octadecadienoic: PUFA 18:2
Octadecatrienoic: PUFA 18:3
Octadecatetraenoic: PUFA 18:4
Eicosatetraenoic: PUFA 20:4
Eicosapentaenoic: PUFA 20:5
Docosapentaenoic: PUFA 22:5
Docosahexaenoic: PUFA 22:6
ARTIEL: Arthritis in older adults
